# Glycation Product Synthesized in Anhydrous Conditions Mimics an Epitope in Epithelial and Mesenchymal Tissues

**DOI:** 10.3390/biomedicines14010196

**Published:** 2026-01-16

**Authors:** Monika Czech, Elżbieta Gamian, Agata Kochman, Marta Woźniak, Emilia Jaskuła, Piotr Ziółkowski, Andrzej Gamian

**Affiliations:** 1 Józef Rostek Regional Hospital, Gamowska 3, 47-400 Racibórz, Poland; monnika.cz@gmail.com; 2 Department of Clinical and Experimental Pathology, Wroclaw Medical University, Marcinkowskiego 1, 50-368 Wroclaw, Poland; egamian@o2.pl (E.G.); ziolkows@int.pl (P.Z.); 3 Department of Pathology, University Hospital Monklands, Monkscourt Ave, Airdrie ML6 0JS, UK; agatakochman@hotmail.co.uk; 4 Hirszfeld Institute of Immunology and Experimental Therapy, Polish Academy of Sciences, Weigla 12, 53-114 Wroclaw, Poland; emilia.jaskula@hirszfeld.pl (E.J.); andrzej.gamian@hirszfeld.pl (A.G.)

**Keywords:** advanced glycation end-products, AGE, human tissues, monoclonal antibody, tumor marker

## Abstract

**Background**: Advanced glycation end-products (AGEs) are formed and deposited in tissues, contributing to various disorders, including diabetes, other metabolic diseases, and aging. A new epitope, AGE10, was identified in human and animal tissues using a monoclonal antibody raised against synthetic melibiose-derived glycation end-products (MAGE), which were synthesized under anhydrous conditions with bovine serum albumin or myoglobin. The biology of the AGE10 epitope, particularly its role in diseases and in cancer tissues, is not well understood. **Methods**: The study was aimed at investigating the immunohistochemical recognition of AGE10 with the MoAb-anti-MAGE antibody. **Results**: Data obtained show that AGE10 is recognized in striated muscles but not in tumors of muscular origin. AGE10 is also stained in both normal and cancerous salivary glands and in adenomas of the large intestine. The staining is cytoplasmic. **Discussion**: Our approach may provide a methodology for cell biology research; AGE10 may be related to an advanced lipoxidation end-product; further investigation of MAGE may clarify disease mechanisms, support the development of novel therapeutic strategies. **Conclusions**: The key finding is that antibodies recognize mainly the epitope in epithelial and some mesenchymal tissues. Thus, the potential for AGE10 as a diagnostic marker is limited. The implications concern the biology of this epitope, the unique tissue distribution, and a role in cellular metabolism.

## 1. Introduction

Advanced glycation end-product (AGE) formation and accumulation in tissues play an important role in diseases related to diabetes and aging processes [1,2]. In diabetes and other metabolic diseases, glycation products are formed and can be deposited in tissues, thereby playing an important role in pathogenesis [3]. Glycation is defined as the process of binding the carbonyl group of reducing sugars or other aldehydes to free amino groups of proteins. Monitoring the levels of accumulated glycation products enables the study of disease pathogenic mechanisms, which is necessary for identifying effective anti-diabetes and anti-sclerosis therapies. Therefore, both the formed AGEs and the cellular pathways activated by them are investigated. Recently, we have reported the synthesis of melibiose-derived glycation end-products, MAGEs (mel-derived AGEs), which mimic a unique epitope present in human and animal tissues [4,5]. The MAGEs were synthesized under anhydrous conditions with bovine serum albumin or myoglobin. The generated mouse anti-MAGE monoclonal antibody (MoAb-anti-MAGE) recognizes a native analogous glycation product in living organisms, tentatively called AGE10 [5]. MAGE cross-reactive auto-antibodies were detected in patients with diabetes [4]. An immunochemical assay to determine the commonly occurring epitope AGE10 was developed based on new synthetic model MAGEs and the monoclonal antibody MoAb-anti-MAGE [4,5]. The presence of this epitope in sera from patients with diabetes was confirmed, and the progression of type II diabetes was found to correlate with the levels of the studied AGE10 [5]. Immunochemical experiments revealed significantly lower levels of circulating AGE10 in patients with Alzheimer’s disease compared with healthy controls [6]. Reduced AGE10 levels were also observed in the sera of patients with allergic rhinitis and chronic Epstein–Barr virus infection at different stages of virus persistence [7]. In both conditions, specific immune complexes AGE10-anti-AGE10 were found and were associated with pathogenicity [6,7]. In addition to tissue accumulation, the formation of serum immune complexes is considered a pathogenic factor contributing to disease mechanisms [6,7]. However, current knowledge about the biology of this epitope remains limited, particularly regarding its involvement in different diseases, including cancer. The aim of the present study was to determine the presence and level of this epitope in human solid tissue by use of the monoclonal MoAb-anti-MAGE antibody and to examine its distribution across different tissues to gain insight into its biology. Immunohistochemical analyses indicated the presence of AGE10 in epithelial and selected mesenchymal tissues. This article is a continuation of research on glycated proteins and their biological activities, including the application of diagnostic markers with tools synthesized under anhydrous conditions.

## 2. Materials and Methods

### 2.1. Materials

All chemicals were acquired from Sigma-Aldrich (St. Louis, MO, USA), unless otherwise stated. The proteins used for glycation were used in the form as purchased, and bovine serum albumin (BSA) (Sigma-Aldrich, Steinheim, Germany) was first purified on a gel filtration column (HW-55S Toyopearl resin) as described in [8]. The following secondary antibodies were used: goat anti-mouse immunoglobulins-HRP (DAKO, Glostrup, Denmark) and goat anti-mouse IgE-HRP (Acris, Herford, Germany). All methods were carried out in accordance with relevant guidelines and regulations. Synthesis of model protein MAGEs, generation of rabbit polyclonal anti-MAGE antibodies, and generation of mouse monoclonal antibody binding MAGEs were described in our previous works [4,5,6]. The reactivity of the obtained monoclonal antibody had the same specificity as the initially used monospecific polyclonal rabbit antibody; therefore, monoclonal antibodies have been used in these experiments.

### 2.2. Methods

#### 2.2.1. Synthesis of Model Protein MAGEs and Low-Molecular-Mass (LMW) MAGEs

**Figure 1:**
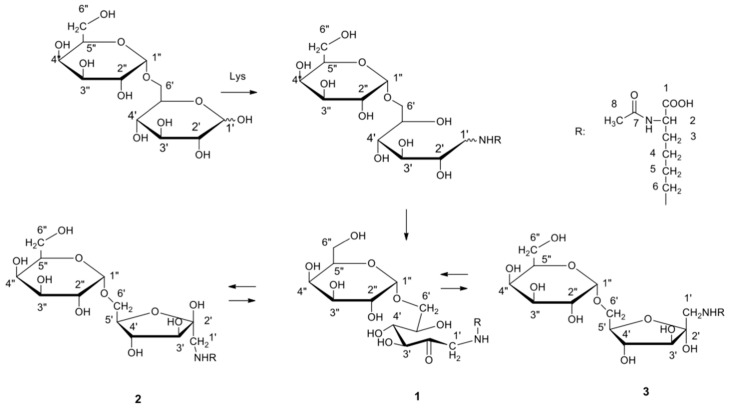
Proposed pathway of microwave glycation in the dry state (MWG), reaction of melibiose with Nα-acetyl lysine leading to fructoselysine, open-chain intermediates, and isomeric forms of novel MAGEs [4].

Protein and low-molecular-mass MAGEs were synthesized as previously described [4]. Briefly, glycation was performed under microwave conditions in the dry state. To generate glycation products, a miliQ water solution mixture containing protein monomer and melibiose (mel; Fluka, St. Louis, MO, USA) was prepared. The solutions were lyophilized and subsequently subjected to a microwave set-up at 85 °C for 45 min to obtain MAGEs. The products formed on proteins were next fractionated on a Sephadex G-200 (Pharmacia, Uppsala, Sweden) column in PBS. The material from the individual fractions was pooled, dialyzed against water, and lyophilized for further characterization. The MAGE product was subsequently used for the production of the monoclonal MoAb-anti-MAGE antibody [4]. Low-molecular-mass MAGEs synthesized for structural study of the molecule mimicking the natural epitope was prepared using a 1:1 mixture of Nα-acetyl-lysine and melibiose dissolved in miliQ water. The mixture was lyophilized and subjected to glycation in a microwave reactor (60 °C) for 25 min. The generated LMW MAGEs were separated on a HW-40S column. The eluent was monitored with the anti-MAGE Ab. The structure of the product established [4] is shown in Figure 1.

#### 2.2.2. Immunohistochemistry

The human samples were collected for research purposes in 2000–2005 and 2017–2020 from the tissue bank of the Department of General Pathology at Wroclaw Medical University after the study protocol was approved by the Bioethics Committee of Wroclaw Medical University, Poland (ST-727), and the study was conducted in accordance with the Helsinki Declaration of 1975, as revised in 1983. Written informed consent was obtained from each patient before tissue collection. The study was also approved by the Bioethics Commission of the Institute of Immunology and Experimental Therapy (KB-3/2024) regarding the use of monoclonal antibodies. Authors had no access to information that could identify individual participants during or after data collection. The experiments were performed as previously described [5]. Antibody specificity is inferred from prior publications [4,5,6,8,9], and confirmatory blocking experiments in tissue sections were not performed in this study.

## 3. Results

Antibodies were studied for their reactivity with antigen present in several human tissue sections. For these experiments, normal, healthy tissues were used, as well as material obtained from rejected renal transplants, primary benign and malignant tumors, and from metastatic carcinoma. In total, 200 tissue samples were studied. Selection was also based on metabolic activity due to some physiological reactivity of muscle tissue, as was reported earlier [4]. This tissue could serve as a reference tissue regarding AGE10. No staining was observed using antibodies on

(1) normal tissues and cells, such cartilage and chondrocytes, fetal tissues, pneumocytes, osteocytes, lymphocytes, splenic lymphatic vessels, and smooth muscle cells of the urinary bladder wall; nervous tissue, decidual cells, vessels of renal glomeruli, and connective tissue cells; adipose tissue cells, ovarian follicular cysts, and rheumatic nodules;
(2) benign tumors, such as uterine myoma or lipoma;
(3) malignant tumors, such as ovarian borderline tumor, lymph nodes in Hodgkin’s disease, small cell carcinoma of the lung, gingival squamous cell carcinoma, atypical endometrial hyperplasia, meningioma, signet ring cell carcinoma of the stomach, synovial sarcoma, Burkitt lymphoma, liposarcoma, and GISTs.

**Figure 2:**
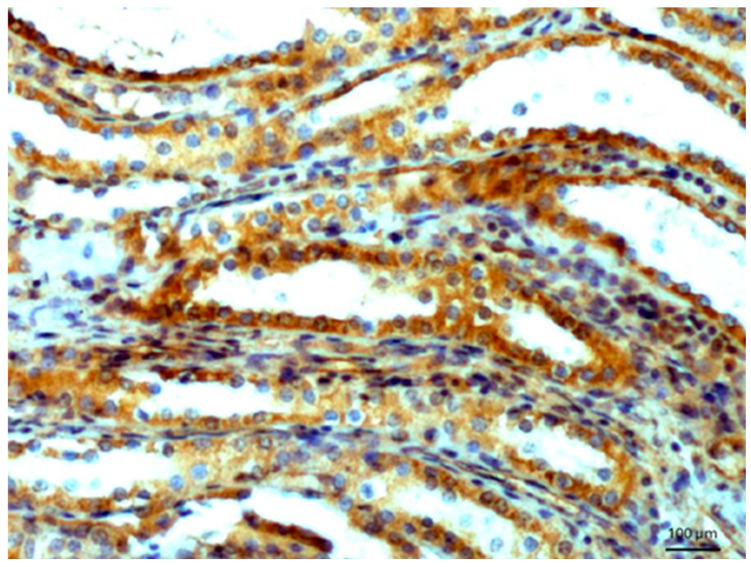
Normal renal tubules. Immunostaining for AGE10, 100×. Positive reaction is clearly seen in the cytoplasm of cells. Counterstained with hematoxylin.

In the rejected renal transplants, immunostaining was observed entirely in the transitional epithelium, while other tissues were not reactive with the antibodies. Normal epithelium of renal tubules also exhibited a positive reaction (Figure 2), while no reactivity was found in the glomeruli. In dysplastic kidney with cysts, a very weak reaction was detected in renal tubules. A positive reaction was observed in the glands of the endometrium.

**Figure 3:**
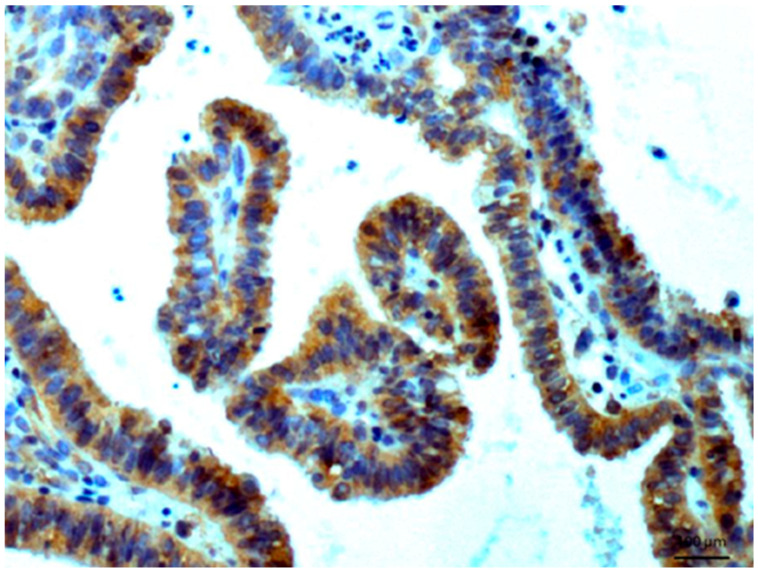
Endometrial adenocarcinoma with formation of papillary structures. Immunostaining for AGE10, 100×. Positive reaction is clearly seen in the cytoplasm of cancer cells. Counterstained with hematoxylin.

**Figure 4:**
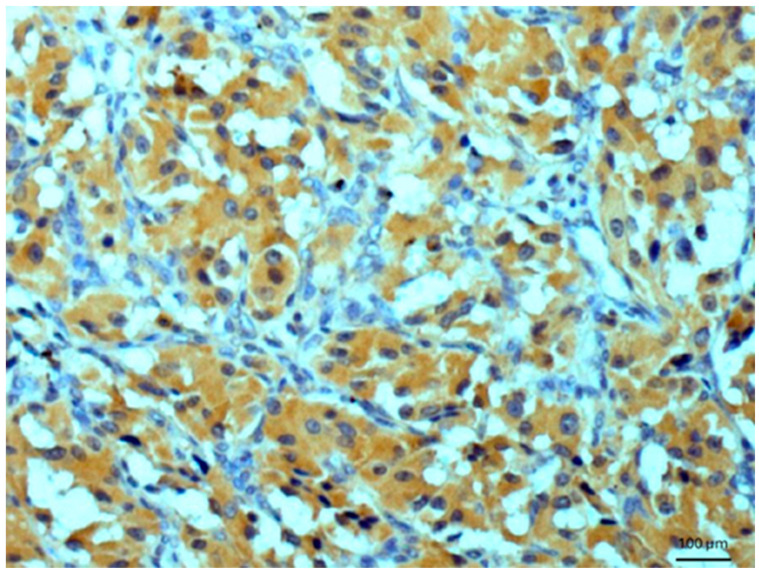
Benign chemodectoma. Immunostaining for AGE10, 100×. Positive reaction is clearly seen in the cytoplasm of tumor cells. Counterstained with hematoxylin.

**Figure 5:**
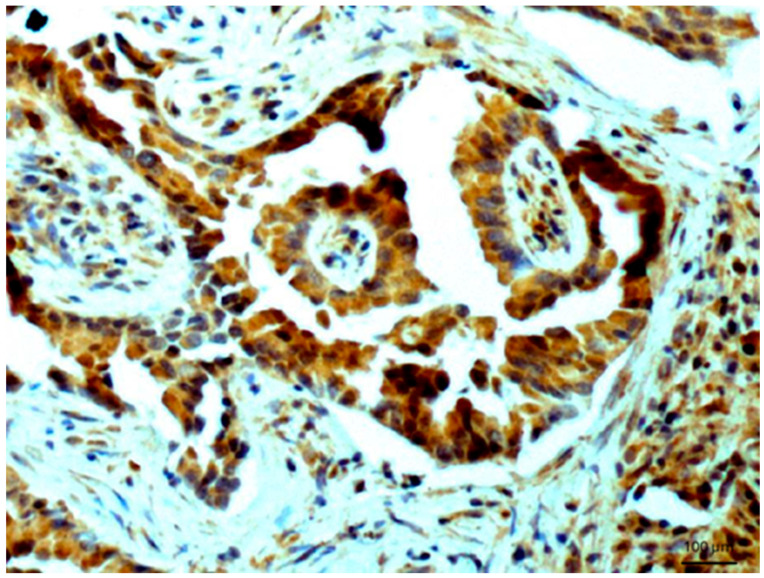
Lung adenocarcinoma. Immunostaining for AGE10, 100×. Positive reaction is clearly seen in the cytoplasm of cancer cells. Counterstained with hematoxylin.

**Figure 6:**
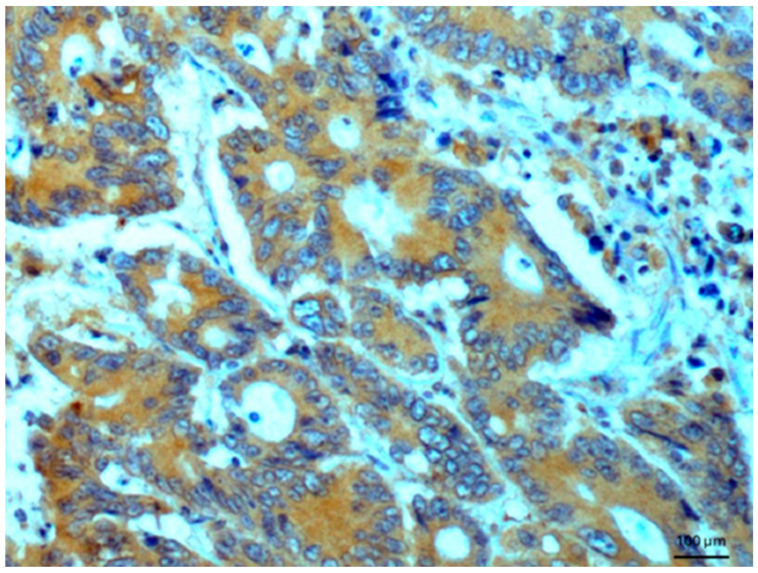
Metastatic colon adenocarcinoma in the liver. Immunostaining for AGE10, 100×. Positive reaction is seen in the cytoplasm of carcinoma cells.

**Figure 7:**
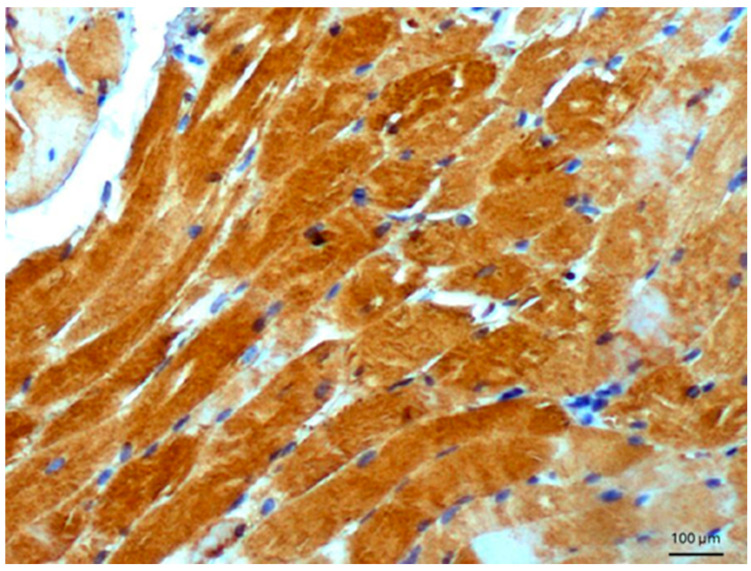
Normal skeletal muscle. Immunostaining for AGE10, 100×. Positive reaction is clearly seen in the cytoplasm of normal muscle cells.

**Figure 8:**
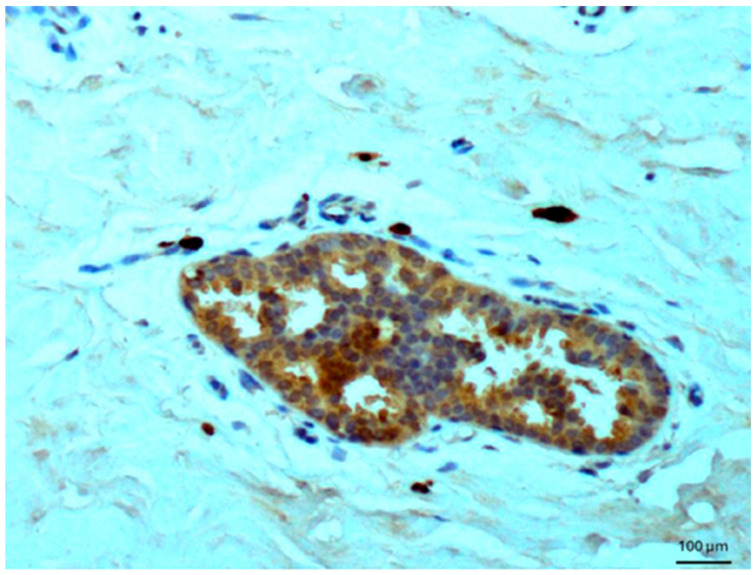
Normal mammary gland. Immunostaining for AGE10, 100×. Positive reaction is clearly seen in the cytoplasm of glandular cells. Counterstained with hematoxylin.

**Figure 9:**
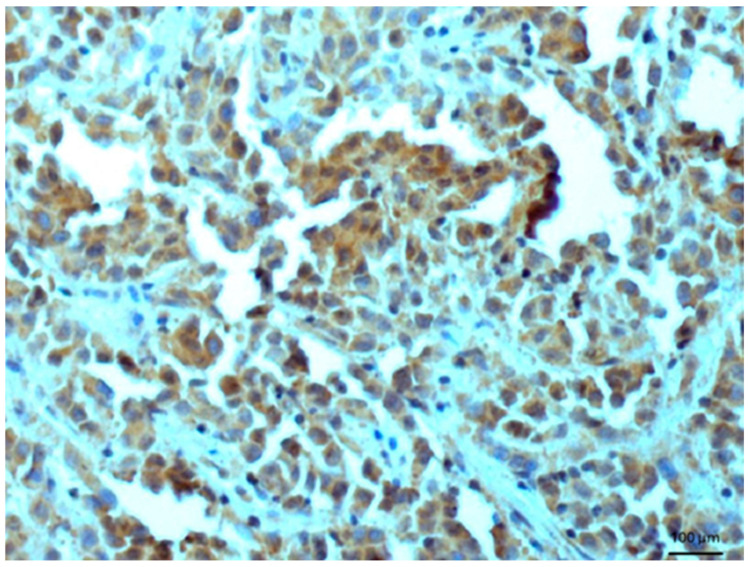
Breast invasive ductal carcinoma of no special type. Immunostaining for AGE10, 100×. Positive reaction is clearly seen in the cytoplasm of cancer cells. Counterstained with hematoxylin.

**Figure 10:**
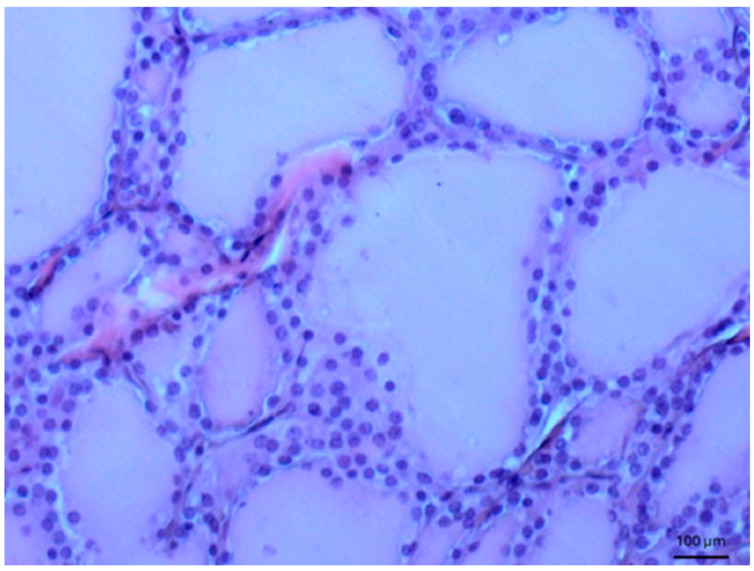
Normal thyroid gland. No staining was found in this case, 100×. Counterstained with hematoxylin.

**Figure 11:**
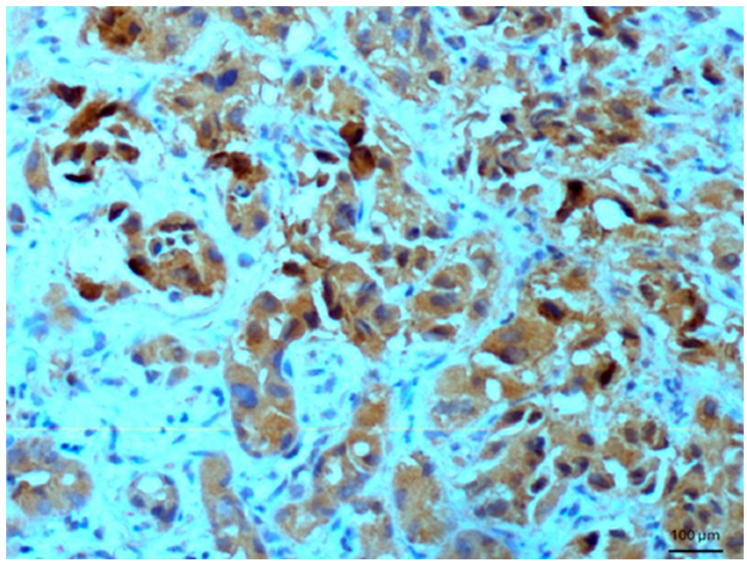
Cholangiocellular carcinoma of the liver. Immunostaining for AGE10, 100×. Positive reaction is clearly seen in the cytoplasm of cancer cells. Counterstained with hematoxylin.

**Figure 12:**
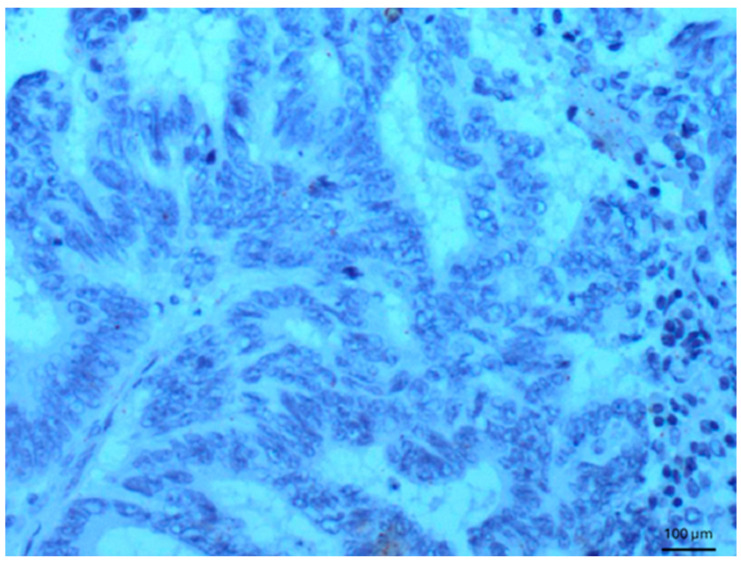
Metastatic colon adenocarcinoma in the liver. Control staining for II antibody, 100×. No reaction was seen in carcinoma cells.

Other tissues, such as normal uterine myometrium, remained unchanged after the immunohistochemical procedure. Endometrial adenocarcinoma cells (Figure 3) and fallopian tube epithelium stained positively. No reaction was observed in atypical endometrial hyperplasia. Decidual cells showed no reactivity. Some benign tumors, such as chemodectoma, also showed positive reactivity (Figure 4), as did various malignant tumors, including pulmonary adenocarcinoma (Figure 5) and metastasizing carcinoma (Figure 6). Positive reaction was observed in skeletal muscle cells (Figure 7). Positive cytoplasmic reactivity was observed in cells of normal breast (Figure 8) and the invasive ductal breast carcinoma (Figure 9), whereas gynecomastia cells remained unstained. Normal thyrocytes presented no reactivity (Figure 10), but thyrocytes in goiter and cells of hyperplastic parathyroid glands stained in the cytoplasm. Oncocytoma cells showed cytoplasmic reactivity. Cells of hepatoblastoma stained in the cytoplasm, and the cells of metastatic carcinoma in the liver also presented a positive reaction. A positive reaction was observed in normal hepatocytes in comparison to the generally weaker staining in hepatocellular carcinoma cells. Figure 11 shows cholangiocellular carcinoma, bile duct carcinoma, and the reaction in the cytoplasm of cancer cells. Representative control staining for II antibody presents metastatic colon adenocarcinoma in the liver, without a reaction in carcinoma cells (Figure 12). Tumor cells of ovarian carcinoma and uterine carcinosarcoma were weakly stained. Serous ovarian borderline tumor stained positively. Askin tumor cells showed a weak cytoplasmic reaction. Positive staining was also observed in cells of the glandular epithelium in a Barrett’s esophagus case. In ganglioneuroma, a benign nervous tissue tumor, positive reactivity in the cell cytoplasm was recorded. In a stomach ulcer in the mucosa, in normal ducts there was a reaction, but no positive reaction was observed in stomach carcinoma infiltration. The post-surgical specimen of stomach signet ring cell carcinoma, including the lymph node with metastatic carcinoma, exhibited no staining in metastatic carcinoma cells. In gastritis, some positive reactivity was noted in the stroma of the gastric epithelium. No reaction was observed in GIST. Pleomorphic rhabdomyosarcoma showed focal positive reactions in tumor cells, due to diffuse necrosis within the tumor. Connective, muscular, and fat tissues within urocystitis were all inflamed, and no reaction was found. Synovial sarcoma cells also showed no staining. Metastatic serous ovarian carcinoma cells stained intensively.

## 4. Discussion

In this study, glycation products in a broad variety of tissues were investigated. Controlling the accumulation of glycation products provides better opportunities to understand disease-inducing mechanisms, and in turn, offers hope for more effective therapies. For this purpose, both the formed AGEs and the cellular pathways they activate were investigated. Studies on glycation products primarily focus on developing reliable tests to determine glycation levels in patients and to better understand the role of AGEs. Conditions for the determination of glycation products in human tissues have been established [9,10]. The preparation of model compounds that mimic those formed in vivo may be helpful in therapy. Synthesized in anhydrous conditions from melibiose, the glycation product MAGE mimics the AGE10 epitope formed in the organism from a non-glucose substrate and may possess a structure different from known AGE structures [4,5]. The epitope represents a new class of glycation products, distinct from classical AGEs (e.g., CML), and further studies are in progress aiming to determine the structure of this new epitope in vivo. The low-molecular-mass AGEs, LMW MAGEs, shown in Figure 1, which inhibit the specific reactivity of anti-MAGE antibodies with AGE10 products, may be applied as inhibitors of tissue glycation reactions or cellular effects induced by glycation through the RAGE receptor. Inhibitors of AGE formation are of great interest for the therapy of metabolic diseases, and studies on new compounds are currently underway [11,12,13].

**Figure 13:**
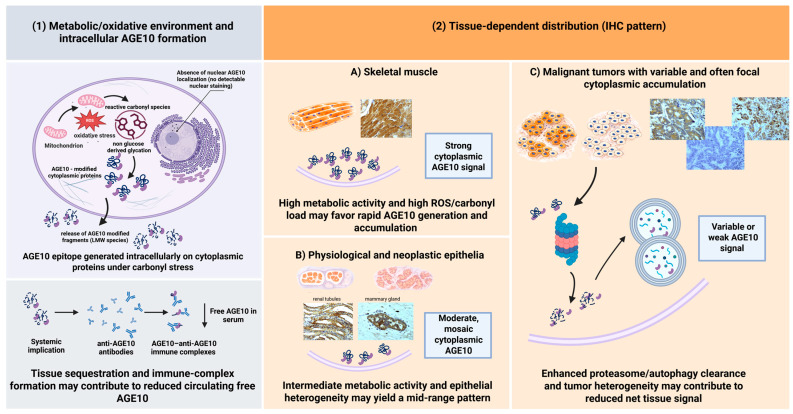
Graphical representation of AGE10 formation (1) and tissue-dependent distribution (2). Working hypothesis integrating metabolic activity, carbonyl stress, and tissue heterogeneity as determinants of intracellular AGE10 immunoreactivity. (1) Metabolic/oxidative environment and intracellular AGE10 formation. The left panel illustrates a proposed intracellular pathway of AGE10 epitope formation under conditions of carbonyl stress. Elevated reactive oxygen species (ROS) and reactive carbonyl species promote non-glucose-derived glycation of cytoplasmic proteins, leading to intracellular generation of AGE10 epitopes. AGE10 immunoreactivity is confined to the cytoplasmic compartment, with no detectable nuclear localization. Proteolytic processing of AGE10-modified proteins may release low-molecular-mass AGE10-containing fragments, which can enter the circulation and form immune complexes with anti-AGE10 antibodies, potentially contributing to reduced levels of free circulating AGE10. (2) Tissue-dependent distribution of AGE10 (immunohistochemical pattern). (**A**) Skeletal muscle. Tissues with high metabolic activity and elevated ROS/carbonyl load, such as skeletal muscle, are hypothesized to favor rapid intracellular AGE10 generation and robust cytoplasmic accumulation, resulting in a strong and diffuse AGE10 immunohistochemical signal. (**B**) Physiological and neoplastic epithelia. Epithelial tissues with intermediate metabolic demand and inherent cellular heterogeneity typically display moderate, mosaic cytoplasmic AGE10 immunoreactivity, yielding a mid-range immunohistochemical pattern. (**C**) Malignant tumors with variable and often focal cytoplasmic accumulation. In malignant tumors, spatial and functional heterogeneity of carcinoma cells, together with enhanced protein turnover and clearance mechanisms, may contribute to variable and often focal AGE10 detectability at the tissue level. Created in https://BioRender.com.

Initial unpublished results on the structure of the tissue epitope indicate some similarities with the synthetic product shown in Figure 1. AGE10 has been detected in immune complexes of sera from patients with Alzheimer’s disease [6] and in patients with allergic rhinitis and chronic Epstein–Barr virus infection [7], as well as in patients with ankylosing spondylitis [14]. Thus, a reduced serum level of AGE10 may result not only from local tissue accumulation, but also from the formation of immune complexes, which could represent a pathogenic factor (Figure 13).

We have shown that antigen AGE10, recognized by specific monoclonal antibodies, is present in a variety of lesions, ranging from mild to malignant, including both epithelial and non-epithelial tissues (Figure 13). It is interesting that, at the physiological level, AGE10 is present in the glandular epithelium of the normal mammary gland. The AGE10 antigen is present in the glandular epithelium, in ductal breast cancer (Figure 9), in endometrial cancer, in bronchial carcinoma, and in bile duct cancer, as well as in normal breast tissue. The observation that rhabdosarcoma, chemodectoma, and cells of normal striated muscle are stained is somewhat unexpected. Strong AGE10 staining in normal skeletal muscle, but absent or focal staining in muscle-derived tumors, indicates biological heterogeneity rather than diagnostic specificity, reinforcing the descriptive nature of the study. Table 1 summarizes the results obtained. It is clear that AGE10 adducts can appear in both pathological and normal cells, and they are also present in mesenchymal structures (muscle, connective tissue, fibrous, cartilaginous, vascular, rhabdosarcoma, chemodectoma, and neuroectodermal origin). A similar pattern was observed in O24 and O56 antigens [15], where reactivity was also present in epithelium and mesenchymal structures. We can distinguish reactivity into an epithelial group (benign, malignant, and metastatic) and a non-epithelial group (muscle, chemodectoma). It remains unclear whether these antibodies can be used in the differential diagnosis of lymph node cancer or epithelial derivatives when other markers are absent. Further research is needed to address this question. Poor reactivity was observed in the prostate glands. Certain malignant tumors, such as squamous cell carcinoma of the gum, endometrial hyperplasia, and prostate (epithelial) cancer, did not exhibit reactivity, while most epithelia did. The reaction was observed in normal gastric ducts, but not in gastric cancer. Variable staining was observed in epithelial malignancies versus benign epithelial tissues. These discrepancies are not fully explained. The absence of quantitative scoring (e.g., H-score or percentage positivity) is a limitation of the study, and future work will address this, as the study aimed at the characterization of a broad set of tissues. At the current stage of research, the origin of the AGE10 antigen in the cell remains unclear, and further research will be undertaken to determine its biosynthesis.

An interesting observation is the positive reactivity in renal tubules, suggesting a possible exchange between the tubules and the external environment. Xue et al. [16] studied the role of advanced glycation end products (AGEs) in exerting their biological effects predominantly through interaction with a specific receptor: the receptor for AGEs (RAGE). This interaction plays a role in the etiology of various diseases, such as diabetes, chronic inflammation, Alzheimer’s disease, and cancer, underscoring the critical importance of the AGE–RAGE pathway in cancer progression and pathology. The study accentuates the therapeutic potential of targeting this pathway in devising treatment modalities [16]. Moreover, Rojas et al. [17] highlighted the role of AGEs accumulation within the extracellular matrix (ECM), which activates receptor-mediated mechanisms for AGEs (RAGE), correlating with several facets of tumor development and growth. This discovery underlines the pivotal function of AGEs in altering the tumor microenvironment, thereby potentially influencing cancer progression and the process of metastasis [17]. Furthermore, Chen et al. reported that AGEs can promote cancer cell proliferation through the stimulation of key transcription factors, providing an additional mechanism by which increased cancer progression may be observed in diabetic patients, reinforcing the notion that hyperglycemia-induced AGEs could further cancer cell growth and malignancy [18]. Similarly, Nam et al. [19] uncovered that AGEs promote the growth and survival of renal cancer cells via the RAGE/Akt/ERK signaling pathways, indicating the AGE–RAGE axis’s contribution to renal cell carcinoma (RCC) growth by promoting PCNA, MMPs, and inhibiting apoptosis. This study highlights the therapeutic promise of inhibiting the AGE–RAGE pathway in renal cell carcinoma treatment strategies [19]. Lastly, Nass et al. [20] identified a positive correlation between the accumulation of specific AGEs, such as carboxymethyl lysine (CML), in breast cancer and estrogen receptor expression, associating high levels of CML with poor prognosis in estrogen receptor-negative cases. This association suggests the potential of CML and possibly other AGEs as prognostic biomarkers for breast cancer, informing treatment decisions regarding tamoxifen or chemotherapy [20]. This insight into the role of AGEs offers a novel avenue for biomarker-driven cancer prognosis and treatment personalization. From an application point of view, it does not matter whether it is mesenchymal or epithelial, normal or cancerous, why AGE10 appears in normal and cancerous tissues. It is difficult to conclude that this could be of diagnostic significance at present, as it is too early to consider AGE10 to be a specific marker.

Nevertheless, we anticipate that our approach may provide a foundational methodology for cell biology research. We hypothesize that AGE10 is related to an advanced lipoxidation end-product. Although our interpretations are not experimentally tested in the current study, the hypotheses direct our future study. Further studies on MAGE may contribute to clarifying disease mechanisms and developing novel therapeutic options for diabetic complications, neuropathology, and cancer.

## 5. Conclusions

In this study, glycation products across a broad variety of tissues were investigated. The glycation product MAGE, synthesized in anhydrous conditions from melibiose, mimics the AGE10 epitope formed in the organism from a non-glucose substrate and may have a structure distinct from known AGE structures. AGE10 is proposed to represent a new class of glycation products. We demonstrated that the AGE10 antigen, recognized by specific monoclonal antibodies, is present in a wide range of lesions, from mild to malignant, including both epithelial and non-epithelial tissues. AGE10 adducts are found in pathological and normal cells, as well as mesenchymal structures, and are localized in the cytoplasm. Results show that AGE10 is detected in striated muscles but not in tumors of muscular origin. It is also present in both normal and cancerous salivary glands and in the adenomas of the large intestine. AGE10 is variably expressed in multiple tissue types, and its diagnostic value is currently uncertain. Antibodies recognize mainly the epitope in epithelial and some mesenchymal tissues.

## Figures and Tables

**Table 1:** Immunohistochemical reactivity of tissues and tumors. Immunohistochemical staining patterns of the analyzed antibodies in normal human tissues, benign lesions, and malignant tumors. Staining intensity is reported as weak, focal, or strong, where applicable. Absence of staining is reported as negative.

Tissue Category	Positive Staining	Negative Staining
Normal tissues	Renal tubules (epithelium); transitional epithelium in rejected renal transplant; endometrial glands; fallopian tube epithelium; skeletal muscle cells; normal breast tissue (cytoplasmic); normal hepatocytes	Cartilage and chondrocytes; fetal tissues; pneumocytes; osteocytes; lymphocytes; splenic lymphatic vessels; smooth muscle cells of the urinary bladder wall; nervous tissue; decidual cells; renal glomeruli; connective tissue cells; adipose tissue cells; ovarian follicular cyst; normal uterine myometrium; normal thyrocytes
Benign/non-malignant lesions	Chemodectoma; ganglioneuroma; goiter (thyrocytes); hyperplastic parathyroid gland; oncocytoma; dysplastic kidney with cysts (weak); gastritis—stromal cells; gastric ulcer—normal ducts	Uterine myoma; lipoma; gynecomastia; rheumatic nodules; gastrointestinal stromal tumor (GIST); urocystitis—connective, muscular, and adipose tissues
Malignant tumors	Endometrial adenocarcinoma; invasive ductal breast carcinoma; pulmonary adenocarcinoma; hepatoblastoma; hepatocellular carcinoma (weaker than normal hepatocytes); cholangiocellular carcinoma (bile duct carcinoma); ovarian carcinoma (weak); metastatic serous ovarian carcinoma (strong); uterine carcinosarcoma (weak); metastasizing carcinoma; Askin tumor (weak); pleomorphic rhabdomyosarcoma (focal); Barrett’s esophagus—glandular epithelium	Small cell lung carcinoma; gingival squamous cell carcinoma; Hodgkin’s disease—lymph nodes; meningioma; signet ring cell carcinoma of the stomach; Burkitt lymphoma; liposarcoma; GIST; metastatic colon adenocarcinoma in the liver (control); gastric carcinoma infiltration; metastatic signet ring cell carcinoma in lymph nodes; synovial sarcoma

## Data Availability

All data generated or analyzed during this study are included in this article.

## Abbreviations

The following abbreviations are used in this manuscript:

AGE	Advanced glycation end-product
MAGE	Melibiose-derived glycation end-product
MWG	Microwave glycation in dry state
RAGE	Receptor for AGEs
